# Stroke Code Improves Intravenous Thrombolysis Administration in Acute Ischemic Stroke

**DOI:** 10.1371/journal.pone.0104862

**Published:** 2014-08-11

**Authors:** Chih-Hao Chen, Sung-Chun Tang, Li-Kai Tsai, Ming-Ju Hsieh, Shin-Joe Yeh, Kuang-Yu Huang, Jiann-Shing Jeng

**Affiliations:** 1 Stroke Center and Department of Neurology, National Taiwan University Hospital, Taipei, Taiwan; 2 Division of Neurology, Department of Internal Medicine, Far-Eastern Memorial Hospital, New Taipei City, Taiwan; 3 Department of Emergency Medicine, National Taiwan University Hospital, Taipei, Taiwan; 4 Graduate Institute of Epidemiology and Preventive Medicine, National Taiwan University, Taipei, Taiwan; University of Münster, Germany

## Abstract

**Background and Purpose:**

Timely intravenous (IV) thrombolysis for acute ischemic stroke is associated with better clinical outcomes. Acute stroke care implemented with “Stroke Code” (SC) may increase IV tissue plasminogen activator (tPA) administration. The present study aimed to investigate the impact of SC on thrombolysis.

**Methods:**

The study period was divided into the “pre-SC era” (January 2006 to July 2010) and “SC era” (August 2010 to July 2013). Demographics, critical times (stroke symptom onset, presentation to the emergency department, neuroimaging, thrombolysis), stroke severity, and clinical outcomes were recorded and compared between the two eras.

**Results:**

During the study period, 5957 patients with acute ischemic stroke were admitted; of these, 1301 (21.8%) arrived at the emergency department within 3 h of stroke onset and 307 (5.2%) received IV-tPA. The number and frequency of IV-tPA treatments for patients with an onset-to-door time of <3 h increased from the pre-SC era (*n* = 91, 13.9%) to the SC era (*n* = 216, 33.3%) (*P*<0.001). SC also improved the efficiency of IV-tPA administration; the median door-to-needle time decreased (88 to 51 min, *P*<0.001) and the percentage of door-to-needle times ≤60 min increased (14.3% to 71.3%, *P*<0.001). The SC era group tended to have more patients with good outcome (modified Rankin Scale ≤2) at discharge (49.5 *vs.* 39.6%, *P = *0.11), with no difference in symptomatic hemorrhage events or in-hospital mortality.

**Conclusion:**

The SC protocol increases the percentage of acute ischemic stroke patients receiving IV-tPA and decreases door-to-needle time.

## Introduction

Worldwide, around 15 million people have a stroke every year, and with about 5 million deaths [1]. It is estimated that one in six people worldwide will have a stroke in there lifetime [2]. Stroke remains one of the leading causes of death and disability even in the developed countries [3], [4]. Of all strokes, about 80% are ischemic. In the setting of acute ischemic stroke management, thrombolysis with intravenous tissue plasminogen activator (IV-tPA) within 3 or 4.5 h of symptom onset is the only treatment proven effective to-date and has been shown to be highly cost-effective and to decrease long-term functional dependence [5], [6].

Despite increased public recognition and promotion in health campaigns, IV-tPA usage among all acute ischemic stroke patients has remained disappointingly low, with reported rates of 3% to 10% in Europe and North America [7]–[9] and only 1.5% in Taiwan [10]. The main barrier to successful thrombolysis is usually a lack of timely administration within the therapeutic window. A pooled analysis of previous thrombolytic trials of acute ischemic stroke revealed that the benefit of IV-tPA decreases as the time from stroke onset to start of treatment increases [11]. Shorter onset to IV-tPA treatment times following stroke is associated with reduced mortality and symptomatic intracranial hemorrhage and with better functional outcome at discharge [12]. To achieve more timely administration of IV-tPA and more successful thrombolytic therapy, earlier hospital presentation of acute stroke patients and a reduction in intra-hospital delays are essential.

Acute stroke care implemented with “Stroke Code” (SC) or similar strategies is reported in several studies to enhance IV-tPA administration and reduce door-to-needle (DTN) time [13]–[27]. Current guidelines of the American Heart Association/American Stroke Association (AHA/ASA) recommend a door-to-computed tomography (CT) completed within 25 min and a DTN time within 60 min [28], [29]. Usually, an establishment of stroke unit, a specialized acute stroke team, and screening criteria for SC activation were required. One study reported a SC program based on the computerized physician order entry system that allowed medical orders to be communicated over a computer network linked to a hospital information system with physicians, nurses, technicians, and other staff in various departments, and the program was successfully implemented this program in 10 hospitals to reduce in-hospital time delay [19]. Another study implemented a protocol with 10 best practices to lower DTN times, and the program was later incorporated into the Target Stroke Initiative launched by the AHA/ASA in 2011 [26], [28]. Some studies even adopted pre-hospital notification programs to increase access to thrombolysis [15], [18], [23]. In the 1,030 Get With The Guidelines (GWTG) – Stroke hospitals including 71,169 acute ischemic stroke patients treated with IV-tPA, the proportion of patients with DTN ≤60 min improved from 26.5% during the preintervention period of 2003–2009 to 41.3% during the postintervention period of 2010–2013 by the Target: Stroke strategies for quality improvement [30]. It therefore seems reasonable to propose that implementation of a multidiscipline coordinated system, such as is associated with SC, across various medical departments in an institute would accelerate the overall process leading to the identification of acute stroke patients eligible for thrombolytic therapy. The present study aimed to investigate the impact of SC on the performance of thrombolytic therapy and on functional outcomes for acute ischemic stroke patients.

## Methods

### Ethics Statement

This study was approved by the Institutional Review Board of National Taiwan University Hospital to prospectively collect information on acute stroke patients, including stroke severity, risk factors, stroke mechanisms, and outcome. All patients gave their written informed consent.

### The “Stroke Code” Protocol

**Figure 1:**
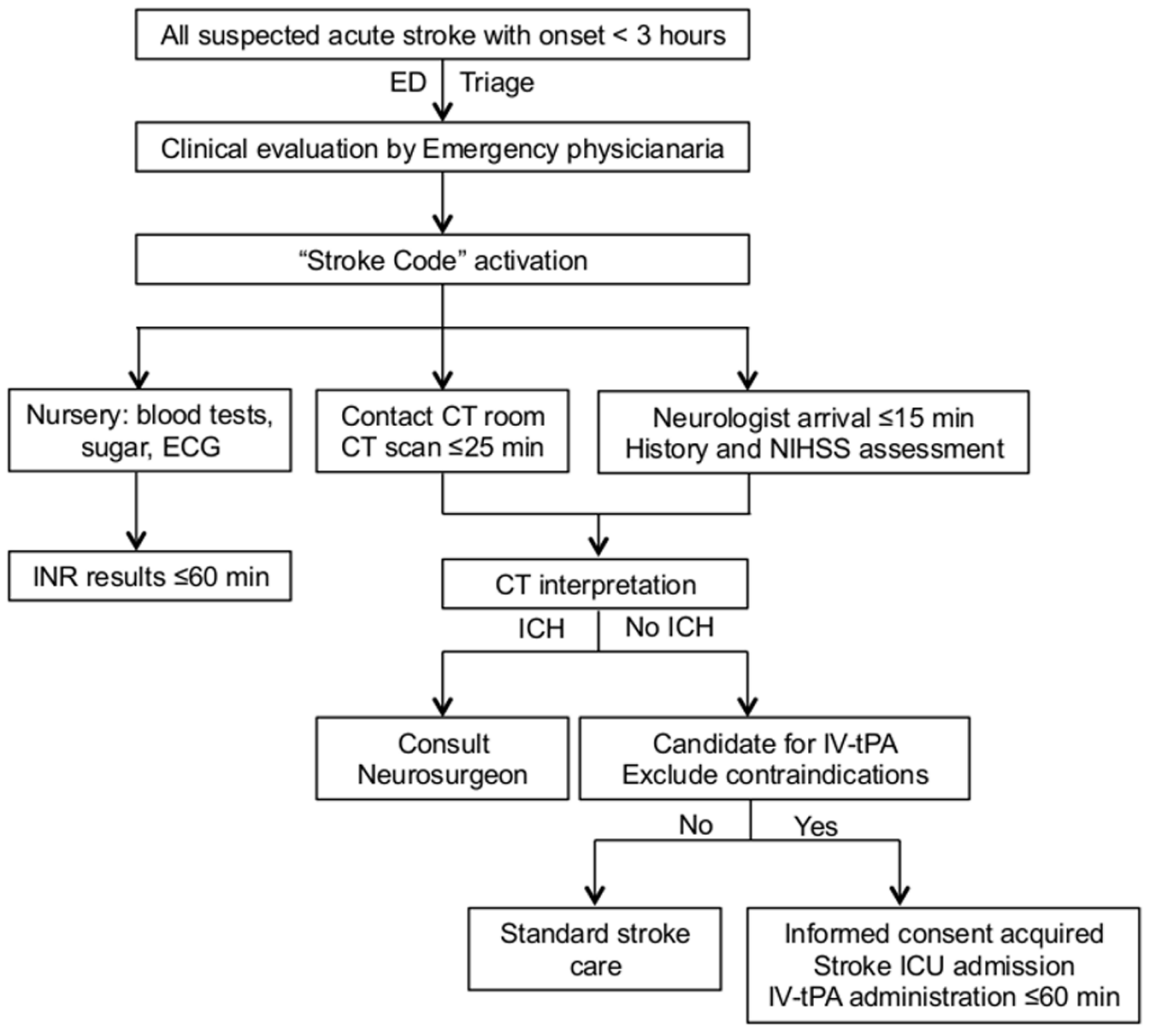
Flowchart of the “Stroke Code” Protocol. Abbreviations: CT, computed tomography; ECG, electrocardiogram; ED, emergency department; ICH, intracranial hemorrhage; INR, international normalized ratio; IV-tPA, intravenous tissue plasminogen activator.

The SC protocol has been employed at the National Taiwan University Hospital since August 2010. The protocol, which is summarized in Figure 1 (and Supporting Information S1), involves the cooperation and integration of the emergency, radiology, laboratory medicine, and neurology staffs that conduct the initial assessment, imaging, and evaluation of acute ischemic stroke patients to expedite acute stroke treatment, particularly with IV-tPA.

When hyperacute stroke is suspected, the triage nurse contacts the physician at the critical care section of the emergency department (ED) to determine whether the SC protocol should be activated immediately. The eligible time window for onset-to-door time was 3 h during the initial period of SC use and was extended to 3.5 h in March 2012 to include potentially treatable patients within the therapeutic window of 4.5 h. Once SC is activated, the patient is triaged to the critical care section, and computer-based text messages are sent simultaneously to the duty radiologist, the consultant neurologist, the stroke nurse practitioner, and the on-call stroke attending staff. The consultant neurologist, who is the duty senior resident, arrives at bedside within 15 min to assess the patient, to confirm the time of onset and the likelihood of acute stroke, and to perform a clinical examination that includes scoring according to the National Institute of Health Stroke Scale (NIHSS). The ED physician contacts the CT room by a hotline and sends the patient for a CT scan as a first priority. The goal is to achieve a door-to-CT time ≤25 min for more than 75% of patients. Meanwhile, the ED nurse performs emergency blood tests, including blood glucose measurements, and an electrocardiogram. A special mark is attached to the tube containing the blood sample for priority examination in the laboratory, particularly with respect to determination of the prothrombin time/international normalized ratio (INR). Because an INR <1.7 is a prerequisite for IV-tPA administration, a target door-to-INR time of ≤60 min is set into the protocol. The CT scan is interpreted by the duty radiologist and confirmed by the neurologist immediately. If no intracranial hemorrhage (ICH) is identifiable by CT scan, the neurologist re-evaluates the eligibility of the patient for IV-tPA based on Taiwan Stroke Society guidelines [31]. The benefits and risks of IV-tPA are explained to the patient or health care proxy. If no absolute contraindication is identified and the patient or health care proxy agrees to IV-tPA treatment, the patient is sent to the stroke intensive care unit immediately. We adopted the NINDS study protocol and Taiwan Stroke Society guidelines for IV-tPA use [5], [31]. Patients are given IV-tPA up to 0.9 mg/kg, to a maximum of 90 mg. Ten percent of the total dose is administered as a bolus while the remainder is given by continuous intravenous infusion over one hour in the stroke unit. The goal is to achieve a DTN time ≤60 min in at least 50% of patients. Stroke educational activities including stroke diagnosis, patient evaluation and NIHSS assessment, acute management and IV-tPA use, and post-thrombolytic care are held for neurology resident, ED personnel and nursing staff. Reports of time intervals and barrier to treatment protocol are discussed with the ED staff on a monthly basis.

### Patients and Data Collection

This study was based on a prospective stroke registry at the National Taiwan University Hospital [32], [33]. All acute ischemic stroke patients who arrived at the ED and were admitted from January 2006 to July 2013 were included. Patients with in-hospital strokes or who were referred from other hospitals after receiving thrombolysis were excluded. Because the “SC” protocol was initiated in August of 2010, the study time period was divided into the “pre-SC era” (January 2006 to July 2010) and the “SC era” (August 2010 to July 2013). Irrespective of SC activation, patients who arrived at the ED within 3 h of stroke onset were identified. Demographic data (age and gender), medical history and vascular risk factors, including hypertension, diabetes mellitus, dyslipidemia, atrial fibrillation, coronary artery disease, prior stroke, and smoking were recorded. Initial stroke severity was assessed by the NIHSS. Acute ischemic stroke patients who received IV-tPA were extracted from the registry and specially encoded in another database. The important time points, included stroke symptom onset (onset time), presentation to the ED (door time), neuroimaging (CT time), laboratory findings (INR time), and bolus IV-tPA administration (needle time), were all specifically recorded by the nursing practitioner. Stroke characteristics and outcomes were recorded. Patients who arrived at the ED during working hours (Monday to Friday, 8 _AM_ to 5 _PM_) were also identified. The stroke subtype was defined according to the Trial of Org 10172 in Acute Stroke Treatment classification [34]. Follow-up brain imaging by CT or magnetic resonance image was routinely performed 24 to 36 h after IV-tPA administration to ascertain whether ICH had occurred; symptomatic ICH was defined as a neurological deterioration (NIHSS ≥2 points) occurring within 36 h and with no radiological findings that might have been responsible for this deterioration other than hemorrhage. Outcomes were assessed by the modified Rankin Scale (mRS) score at discharge and at 3 months after stroke, and a good outcome was defined as mRS ≤2.

### Statistical Analysis

We used frequencies with percentages to describe discrete variables, and means ± SD or medians (interquartile range) to describe continuous variables. Patients who received IV-tPA in the “pre-SC era” and “SC era” were compared according to their clinical characteristics, time intervals, and outcome. For continuous variables, we used independent sample t test for age and Mann-Whitney *U*-test for initial NIHSS and time intervals. Either chi-squared test or Fisher’s exact test were used for categorical variables. Multivariable logistic regression analysis models were used to assess the predictors of good outcome (mRS ≤2) at discharge and 3 months after stroke, and DTN time ≤60 min. Factors entered the analysis models were those being significant in the univariate analysis and those considered clinical relevant. A *P* value <0.05 was considered to indicate statistical significance. Statistical analysis was performed using the SPSS software package version 20.0 (SPSS Inc., Chicago, IL, USA).

## Results

From January 2006 to July 2013, 5957 patients with acute ischemic stroke were admitted; of these, 1301 (21.8%) arrived at the ED within 3 h of stroke onset and 307 (5.2%) received IV-tPA. Of the 307 patients (median age, 69 years; male, 59%) receiving IV-tPA, 91 were admitted during the pre-SC era and 216 were admitted during the SC era. The frequency of IV-tPA usage increased significantly during the SC as compared to the pre-SC era; rates for all acute ischemic stroke patients (8.6% *vs.* 2.6%, respectively; *P*<0.001) and for those arriving at the ED within 3 h (33.3% *vs.* 13.9%, respectively; *P*<0.001) were both increased. During the SC era, SC was activated for 1143 patients at an average of 31.8 patients per month; activations included 865 codes for acute stroke and 278 codes for stroke-mimic. Overall, 216 (18.9%) of the 1143 SC patients received IV-tPA, with an average of one IV-tPA administration per 5.3 codes.

Demographic variables and stroke subtypes were comparable between the two eras, excepting that more patients with atrial fibrillations were admitted during the pre-SC era and more patients with dyslipidemia were admitted during the SC era (Table 1). Additionally, the median initial NIHSS score was higher in the pre-SC era. The median onset-to-door time was 45 min for the pre-SC era and 58 min for the SC era (*P = *0.011). The median door-to-CT time was 24 min for the pre-SC era and 11 min for the SC era (a 13-min or 54% reduction, *P*<0.001). The median DTN time was 88 min for the pre-SC era and 51 min for the SC era (a 37-min or 42% reduction, *P*<0.001). The median onset-to-needle time was also reduced (145 min for the pre-SC era and 125 min for the SC era; *P*<0.001). During the SC era, 91.7% of cases reached the door-to-CT goal of ≤25 min and 71.3% reached the DTN goal of ≤60 min whereas during the pre-SC era, 52.7% and 14.3% of cases reached these respective goals. The observed differences in goal achievements between the two eras were statistically significant (both *P*<0.001).

**Table 1:** Comparison of Demographics, Time Interval, and Outcome Variables Between the Pre-Stroke Code and Stroke Code Eras.

	Pre-Stroke Code era (n = 91)	Stroke Code era (n = 216)	*P* value
Age, years	68.6±12.0	67.6±12.7	0.497
Male	59 (64.8)	122 (56.5)	0.174
*Stroke risk factors*			
Hypertension	67 (73.6)	159 (73.6)	0.998
Diabetes mellitus	28 (30.8)	71 (32.9)	0.719
Dyslipidemia	22 (24.2)	85 (39.5)	***0.010***
Atrial fibrillation	55 (60.4)	100 (46.3)	***0.024***
Coronary artery disease	14 (15.4)	40 (18.5)	0.510
Prior stroke	15 (16.5)	48 (22.2)	0.256
Smoking	20 (22.0)	53 (24.5)	0.631
Initial NIHSS (IQR)	15 (10–21)	12 (7–17)	***<0.001***
*Stroke subtype*			
Cardioembolism	54 (59.3)	105 (48.6)	0.324
Large-artery atherosclerosis	20 (22.0)	52 (24.1)	
Small-vessel occlusion	5 (5.5)	18 (8.3)	
Others	12 (13.2)	41 (19.0)	
Arrival during working hours	39 (42.9)	72 (33.3)	0.113
*Time interval, min (IQR)*			
Onset-to-door	45 (30–65)	58 (32–94.5)	0.009
Door-to-CT	24 (19–38.5)	11 (9–13)	***<0.001***
Door-to-INR	60 (49–119)	43 (37–52.3)	***<0.001***
CT-to-needle	61 (44–79)	40 (32–51)	***<0.001***
Door-to-needle	**88 (67–107)**	**51 (43–64)**	***<0.001***
Onset-to-needle	145 (122–163)	125 (90.3–157)	***<0.001***
Door-to-CT ≤25 minutes	48 (52.7)	198 (**91.7**)	***<0.001***
Door-to-needle ≤60 minutes	13 (14.3)	154 (**71.3**)	***<0.001***
Onset-to-needle 3–4.5 hours	7 (7.7)	23 (10.6)	0.426
*Outcome*			
Symptomatic ICH	7 (7.7)	10 (4.6)	0.285
Good outcome at discharge	36 (39.6)	107 (49.5)	0.110
Good outcome at 3 months	40 (44.0)	109 (50.5)	0.298
In-hospital mortality	6 (6.6)	7 (3.2)	0.216

CT, computed tomography; ED, emergency department; EMS, emergency medical service; ICH, intracranial hemorrhage; INR, international normalized ratio; IQR, interquartile range.

The SC era group tended to include more patients with good outcome (mRS ≤2) at discharge than did the pre-SC era group (49.5 *vs.* 39.6%, *P = *0.110) and at 3 months after stroke (50.5% *vs.* 44.0%, *P = *0.298) and with less symptomatic ICH (4.6% *vs.* 7.7%, *P = *0.285) and in-hospital mortality (3.2% *vs.* 6.6%, *P = *0.216). However, these comparisons did not reach significance. In the multivariate analysis of factors related to good outcome at 3 months after stroke, only age and initial NIHSS score were found to be significant (both *P*<0.01, Table 2).

**Table 2:** Multivariate Analysis of Good Outcome (mRS ≤2) at 3 Months.

	Unadjusted OR (95% CI)	*P* value	Adjusted OR (95%) CI*	*P* value
Age	0.94 (0.92–0.96)	<0.001	0.95 (0.92–0.97)	<0.001
Male sex	1.74 (1.10–2.76)	0.019	1.38 (0.80–2.37)	0.248
Initial NIHSS	0.85 (0.82–0.89)	<0.001	0.86 (0.82–0.90)	<0.001
Atrial fibrillation	0.40 (0.25–0.64)	<0.001	0.87 (0.50–1.50)	0.607
DTN ≤60 minutes	1.52 (0.97–2.39)	0.069	1.07 (0.63–1.83)	0.796
“Stroke Code” use	1.30 (0.79–2.13)	0.298	–	

*Variables entered for analysis: Age, sex, Initial NIHSS, atrial fibrillation, DTN ≤60 minutes.

CI, confidence interval; DTN, door-to-needle; NIHSS, National Institute of Health stroke scale; OR, odds ratio.

When factors associated with a DTN ≤60 min were examined, age (OR = 0.98 for every year increase, *P = *0.026) and onset-to-door time (OR = 1.31 for every 15-min increase, *P*<0.001) remained independent after multivariable adjustment (Table 3). Results were similar when findings were analyzed within the SC era, except that an additional factor of arrival during working hours was observed (OR = 2.06, *P = *0.044).

**Table 3:** Multivariate Analysis of Factors Associated with a DTN ≤60 Minutes.

	All patients		Stroke Code era	
	UnadjustedOR (95% CI)	Adjusted*OR (95% CI)	UnadjustedOR (95% CI)	AdjustedOR (95% CI)
Age	0.98 (0.96–1.00)†	0.98 (0.96–1.00)†	0.97 (0.94–0.99)‡	0.96 (0.94–0.99)‡
Male sex	0.83 (0.54–1.31)	0.65 (0.39–1.07)	0.91 (0.50–1.66)	0.71 (0.37–1.37)
Initial NIHSS	0.95 (0.92–0.99)‡	0.97 (0.94–1.01)	0.97 (0.93–1.02)	1.00 (0.95–1.05)
Onset-to-door time, per 15-minute increase	1.31 (1.19–1.44)‡	1.31 (1.18–1.45)‡	1.23 (1.09–1.38)‡	1.27 (1.11–1.44)‡
Working hours arrival	1.23 (0.77–1.97)	1.34 (0.81–2.23)	1.84 (0.95–3.59)	2.06 (1.02–4.17)†

*Adjusted for age, male sex, initial NIHSS, onset-to-door time, working hours arrival.

† *P*<0.05;

‡ *P*<0.01.

Patients in the SC era were further analyzed with respect to arrival at the ED during working *vs.* non-working hours. More SC activations were observed in the non-working (*n* = 144) than in the working (*n = *72) hours group. The working hours group tended to have a shorter onset-to-door time (53 *vs.* 60 min, *P = *0.280), longer door-to-CT time (12 *vs.* 10 min, *P*<0.001), longer door-to-INR time (45.5 *vs.* 42 min, *P = *0.068), shorter DTN time (49 *vs.* 52 min, *P = *0.142), and shorter onset-to-needle time (112.5 *vs.* 128.5 min, *P = *0.093) (Table S1). Overall outcomes tended to be better for the working hours as compared to the non-working hours group but the difference was not statistically significant.

## Discussion

Findings of the present study reveal that implementation of SC significantly increases IV-tPA administration and significantly shortens DTN time. SC usage was associated with a 13-min reduction in door-to-CT time, a 37-min reduction in DTN time, a 5-fold increase in the percentage of patients reaching the DTN goal of ≤60 min, and a trend toward better functional outcomes without increases in symptomatic ICH or mortality rate. During the SC era, rates for IV-tPA usage among all acute ischemic stroke patients and for an onset-to-door time of <3 h were 8.6% and 33.3%, respectively. These percentages were much higher than those found by a multi-hospital survey conducted in Taiwan during the period of 2006 to 2008 in which only a 1.5% thrombolysis rate was observed for all acute ischemic stroke patients [10]. Thrombolysis rates are usually higher in single center registries than in national registries, with rates ranging from 10.3% to 58% among hyperacute stroke patients (<3 or 4.5 h) [24]. The thrombolysis rate observed in the present study was comparable to most rates reported by single center studies [24]. The SC protocol currently used at the National Taiwan University Hospital is mainly an in-hospital system. Incorporation of pre-notification by the EMS into the protocol, as is recommended by the American Stroke Association [35], should serve to reduce pre-hospital delays. Pre-hospital notification by the EMS was reported in a single center study to reduce DTN times up to 20 min [18] and in the Get With The Guidelines (GWTG) – Stroke by 2 min (78 *vs.* 80 min, *P*<0.0001) [23]. Nonetheless, the proportion of patients arriving within 3 h was only 21.8% in the present study, indicating that the majority of patients were ineligible for IV-tPA. Further public campaign promotions targeted toward early stroke recognition remains indispensable.

The significant decreases in door-to-CT and DTN times, as well as the high proportion of DTN times of ≤60 min, observed during the SC era strongly support the applicability of the SC protocol described in the present study. The median DTN time (51 min) was shorter than that reported by the Safe Implementation of Thrombolysis in Stroke-MOnitoring STudy (SITS-MOST) study (68 min) [36] and was comparable to times reported by selected medical centers [16]–[18], [22], [24]–[26], [37]. The proportions of door-to-CT times of ≤25 min and DTN times of ≤60 min in the present study were 92% and 71% respectively. These values were also slightly better than those observed in a recent report from the Massachusetts General Hospital (69% and 70% respectively) [26].

The shorter DTN time observed for the SC era group compensated for the longer onset-to-door time seen for this group; overall, the onset-to-needle time for the SC era group was 20 min shorter than that for the pre-SC era group. This finding supports the proposal that more patients with longer pre-hospital delays may still be eligible to receive IV-tPA when the smooth DTN process described in this report is employed; such patients include those with an onset time of <3.5 h and who could be treated within 4.5 h. The shorter time window for decision-making by the stroke experts did not offset patient safety, as evidenced by the trend toward less symptomatic ICH and better outcome in the SC era group.

Functional outcome in the SC era group tended to be better than that in the pre-SC era group, although the difference in outcome was not statistically significant. Findings were consistent with those of several other studies in which age and initial stroke severity were found to be strong outcome predictors [38]–[40]. The observation of the present study that SC usage resulted in a 20-min (13.8%) reduction in onset-to-needle time without a statistically significant improvement in outcome may be related to underpowered patient number. Nonetheless, the borderline association between good outcome and DTN time of ≤60 min reported here strengthens the observation that earlier treatment is associated with greater neurological improvement [11].

**Figure 2:**
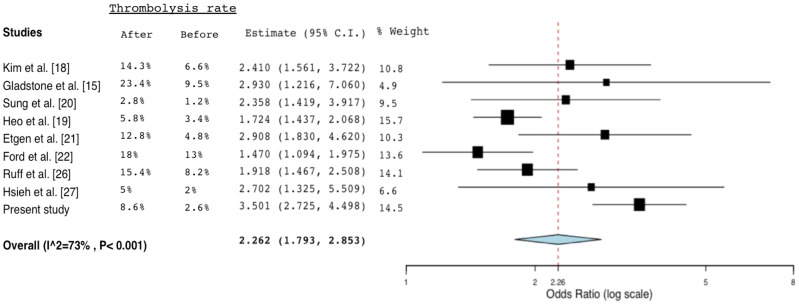
Forest plot of odds ratio for thrombolysis rate among selected studies (after: post-intervention period; before: pre-intervention period). Abbreviation: C.I., confidence intervals.

**Figure 3:**
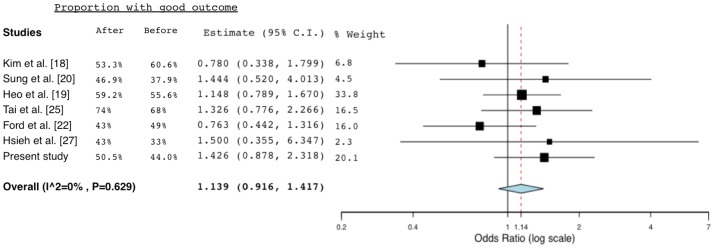
Forest plot of odds ratio for good outcome (mRS ≤2) among selected studies (after: post-intervention period; before: pre-intervention period). Abbreviation: C.I., confidence intervals.

In recent years, there were several studies evaluating the effects of similar implemented programs on the performance of thrombolysis. Those studies with a clear-cut comparison of the thrombolytic rate or functional outcome before and after the intervention, alone with the present study, were summarized in Table S2 (for the methods and results of the literature review, please referred to the Supporting Information S2) [15], [18]–[22], [25]–[27]. A meta-analysis of these studies demonstrated a two-fold increase in chance of thrombolysis rate after the intervention (Figure 2). However, there was only a statistically nonsignificant trend toward good outcome (mRS ≤2) after meta-analysis of the studies with available data (Figure 3). It might suggest that the SC or similar intervention were mainly strategies to enhance thrombolysis administration and hasten DTN time, yet others factors such as age and initial stroke severity contributed to the outcome more directly.

In the present study, the significant determinants of DTN time of ≤60 min for the SC group were younger age and longer onset-to-door time. In the GWTG–Stroke study, predictors of DTN time of ≤60 min included greater initial neurological deficits, on-hours arrival, longer onset-to-arrival time, younger age, male gender, and without atrial fibrillation, diabetes mellitus or prior stroke history [9]. Regarding the age determinant identified in the present study and in the GWTG-Stroke study, it is proposed that longer times may result from the weighting by both patient/family and physician of the benefits *vs.* risks of IV-tPA administration to older patients. Regarding the determinant of DTN time of ≤60 min identified in both studies, it is conceivable that onset-to-door time influences the speed of treatment. For example, patients with longer onset-to-door times may receive faster treatments whereas patients with shorter onset-to-door times may be handled in a more relaxed fashion. The inverse nature of the relationship between onset-to-door time and DTN time was reported to be minimized by implementation of a computerized in-hospital alert system [19]. Findings of the present study support that observation. The correlation efficiency between onset-to-door time and DTN time was found to improve from −0.414 in the pre-SC era to −0.237 in the SC era, reflecting a positive impact of SC on the consistent care of acute stroke patients.

Several studies have found the stroke “weekend effect” that the mortality tended to be higher among ischemic stroke patients admitted on weekends [41]–[46]. The phenomenon was possibly attributed to reduced staffing levels, a decreased availability of resources and diminished access to subspecialty care on weekends. In our protocol, the round-the-clock hospital-based consultant neurologist, the hotline between the ED and CT room, the special mark for the on the blood sample tube, as well as the well-experienced stroke intensive care unit were all strategies to minimize the “weekend effect”. Thus, the overall DTN times and functional outcome were comparable between the working hours and non-working hours group. The findings in our results echoed with a previous large cohort study showing that the establishment of comprehensive stroke centers could reduce the “weekend effect” in the ischemic stroke patients [47].

In most hospitals, IV-tPA was administered at the ED. However in the protocol described in the present report, IV-tPA was administered in the stroke intensive care unit. The advantages of administering IV-tPA in the stroke intensive care unit included a standardized intensive care unit medical bed for precise body weight measurement, rapid access to IV-tPA stored in the intensive care unit, sufficient numbers of skilled nurses familiar with IV-tPA bolus and infusion protocols, close monitoring and management of blood pressure, and availability of bedside carotid duplex and transcranial color-coded sonography to assist in diagnosis of such conditions as large artery occlusion or dissection. The findings that the DTN time observed in the present study was not inferior to those reported at other medical centers and that 71.3% of patients reached the DTN goal of ≤60 min provides assurance that the SC protocol described here is feasible with no additional transportation delays.

Some noteworthy limitations to this study should be mentioned. First, this study was conducted in a single tertiary medical center with sufficient patient volume, experienced physicians trained in stroke management, and 24-h neurology resident coverage. Thus the generalizability of the findings may be limited. Second, a before-after comparison cannot exclude the possibility of other external factors such as a learning effect. However, the observed 3-fold increase in IV-tPA administration rate and the 5-fold increase in the target DTN time of ≤60 min largely preclude other influences. Third, point-of-care INR testing has been proven to accelerate thrombolysis for patients using oral anticoagulants [48]. Although point-of-care testing was adopted at the National Taiwan University Hospital as an auxiliary method in October 2011, testing was not used for every patient. Therefore, further investigation is needed to evaluate the impact of SC on door-to-INR time.

In conclusion, implementation of the “SC” protocol increases the rate of administration of IV-tPA to patients with acute ischemic stroke and decreases DTN time for these patients. Findings support improvement in outcome with no increase in the incidence of symptomatic ICH or in-hospital mortality. Future goals include incorporation of the pre-hospital EMS notification system into the protocol and improvement in execution of the protocol across business hours.

## Supporting Information

Table S1 Comparison Between Stroke Code Patients Arrived During the Working and Non-working Hours.
(DOC)

Table S2 Summary of studies evaluating the performance of thrombolysis before and after strategies implementation.
(DOC)

Supporting Information S1 National Taiwan University Hospital Acute Stroke Protocol.
(DOCX)

Supporting Information S2 Literature Review and Meta-analysis.
(DOCX)

Raw Data S1 (SAV)
