# Supplementary material for: Stroke Code Improves Intravenous Thrombolysis Administration in Acute Ischemic Stroke
Source: PLoS One. 2014 Aug 11;9(8):e104862. doi: 10.1371/journal.pone.0104862 (PMC4128738; doi:10.1371/journal.pone.0104862)
Supplement: Table S1 — Comparison Between Stroke Code Patients Arrived During the Working and Non-working Hours. (DOC) [file pone.0104862.s001.doc]

**Table S1. Comparison Between Stroke Code Patients Arrived During the Working and Non-working Hours**

|  | Non-working hours (n=144) | Working hours (n=72) | *P* value |
| --- | --- | --- | --- |
| Age, years | 67.8 ± 12.5 | 67.2 ± 13.1 | 0.757 |
| Male, % | 85 (59.0) | 37 (51.4) | 0.286 |
| Initial NIHSS (IQR) | 14 (8–18) | 12 (7–19) | 0.412 |
| *Time interval, min (IQR)* |  |  |  |
| Onset-to-door | 60 (33.3–102.3) | 53 (30–92.8) | 0.280 |
| Door-to-CT | 10 (8–12) | 12 (9.5–13) | 0.003 |
| Door-to-INR | 42 (36.3 – 51) | 45.5 (38.5 – 63.3) | 0.068 |
| CT-to-needle | 42 (33–55.5) | 35 (30.5–46) | 0.014 |
| Door-to-needle | 52 (43–65) | 49 (42–58.8) | 0.142 |
| Onset-to-needle | 128.5 (95.5–162) | 112.5 (83–144.8) | 0.093 |
| Door-to-CT ≤25 minutes | 134 (93.1) | 64 (88.9) | 0.296 |
| Door-to-needle ≤60 minutes | 97 (67.4) | 57 (79.2) | 0.071 |
| *Outcome, n (%)* |  |  |  |
| Any ICH | 34 (23.6) | 16 (22.2) | 0.820 |
| Symptomatic ICH | 9 (6.2) | 1 (1.4) | 0.170 |
| In-hospital mortality | 7 (4.9) | 0 (0.0) | 0.098 |
| Good outcome at discharge | 67 (46.5) | 40 (55.6) | 0.211 |
| Good outcome at 3 months | 68 (47.2) | 41 (56.9) | 0.178 |

CT, computed tomography; ED, emergency deparment; ICH, intracranial hemorrhage; INR, international normalized ratio; IQR, interquartile range.
