# Supplementary material for: Stroke Code Improves Intravenous Thrombolysis Administration in Acute Ischemic Stroke
Source: PLoS One. 2014 Aug 11;9(8):e104862. doi: 10.1371/journal.pone.0104862 (PMC4128738; doi:10.1371/journal.pone.0104862)
Supplement: Table S2 — Summary of studies evaluating the performance of thrombolysis before and after strategies implementation. (DOC) [file pone.0104862.s002.doc]

**Table S2.** Summary of studies evaluating the performance of thrombolysis before and after strategies implementation

| Study | Intervention | Study Period | IV-tPA number | Thrombolysis rate | Door-to-CT time (min) | DTN time (min) | Onset-to-needle time (min) | DTN ≤60 min | Good outcome (mRS ≤2) | Symptomatic ICH rate |
| --- | --- | --- | --- | --- | --- | --- | --- | --- | --- | --- |
| Kim *et al.*, 2009 [18] | Pre-hospital notification | 01/2006 ~ 10/2006 | 44 | 6.6% | na | 48.6 | 118.5 | na | 60.6% | 4.9% |
|  |  | 11/2006 ~ 05/2007 | 47 | 14.3% | na | 34.8 | 144.2 | na | 53.3% | 10.0% |
| Gladstone *et al.*, 2009 [15] | Citywide prehospital acute stroke activation program | 02/2004 ~ 06/2004 | 7 | 9.5% | na | 128 | 195 | na | na | na |
|  |  | 02/2005 ~ 06/2005 | 30 | 23.4% | na | 83 | 141 | na | 28% | 10% |
| Sung *et al.*, 2010 [20] | Primary stroke center and acute stroke team | 01/2004 ~ 10/2007 | 29 | 1.2% | 21 | 85 | 128 | na | 37.9% | 6.9% |
|  |  | 11/2007 ~ 06/2009 | 32 | 2.8% | 16 | 67 | 110 | na | 46.9% | 12.5% |
| Heo *et al.*, 2010 [19] | CPOE-based program (10 hospitals) | 1 year before | 199 | 3.4% | 24.7 | 71.7 | 133.6 | na | 55.6% | na |
|  |  | 1 year after | 312 | 5.8% | 17 | 56.6 | 123.8 | na | 59.2% | na |
| Etgen *et al.*, 2011 [21] | Stroke unit | 2005 ~ 2006 | 24 | 4.8% | 10.3 | 62.2 | na | na | na | 4.2% |
|  |  | 2007 ~ 2008 | 95 | 12.8% | 10.4 | 38.5 | na | na | na | 2.1% |
| Tai *et al.*, 2011 [25] | Code stroke | 01/2003 ~ 06/2007 | 96 | 3.9~8.2% | 42 | 90 | 160 | na | 68% | 5% |
|  |  | 07/2007 ~ 12/2010 | 189 | 12.2~17.3% | 23 | 72 | 160 | na | 74% | 7% |
| Ford *et al.*, 2012 [22] | Value stream analysis | 01/2009 ~ 02/2011 | 132 | 13% | 16 | 60 | 131 | 52% | 49% | 3.0% |
|  |  | 03/2011 ~ 03/2012 | 87 | 18% | 1 | 39 | 111 | 78% | 43% | 3.4% |
| Ruff *et al.*, 2014 [26] | MGH stroke protocol | 2003 ~ 2006 | 111 | 8.2% | 30 | 70 | 124 | 32.1% | na | na |
|  |  | 2008 ~ 2011 | 130 | 15.4% | 18 | 47 | 105 | 70.3% | na | na |
| Hsieh *et al.*, 2014 [27] | Video-assisted therapeutic risk communication | 2009 ~ 2011 | 18 | 2% | 32 | 93 | 154 | 11% | 33% | 17% |
|  |  | 2012 | 14 | 5% | 20 | 57 | 136 | 57% | 43% | 21% |
| Present study | Stroke code protocol | 01/2006 ~ 07/2010 | 91 | 2.6% | 24 | 88 | 145 | 14.3% | 44.0% | 7.7% |
|  |  | 08/2010 ~ 07/2013 | 216 | 8.6% | 11 | 51 | 125 | 71.3% | 50.5% | 4.6% |

CPOE, computerized physician order entry; CT, computed tomography; DTN, door-to-needle; ICH, intracranial hemorrhage; IV-tPA, intravenous tissue plasminogen activator; MGH, Massachusetts General Hospital; mRS, modified Rankin scale
