# Supplementary material for: Stroke Code Improves Intravenous Thrombolysis Administration in Acute Ischemic Stroke
Source: PLoS One. 2014 Aug 11;9(8):e104862. doi: 10.1371/journal.pone.0104862 (PMC4128738; doi:10.1371/journal.pone.0104862)
Supplement: Supporting Information S1 — National Taiwan University Hospital Acute Stroke Protocol. (DOCX) [file pone.0104862.s003.docx]

**National Taiwan University Hospital Acute Stroke Protocol**

**Purpose:** To activate the Acute Stroke Protocol for all patients who were last seen well less than 3 hours before Emergency Department (ED) arrival with acute neurological symptoms, in order to carry out necessary managements and possible thrombolysis therapy, and to improve the outcome of acute ischemic stroke patients.

**Suitable personnel:** DTN (Door-to-needle) team members, including the personnel in the Emergency department, Neurology department, Department of Medical Imaging, and Department of Laboratory Medicine.

**Contents of the protocol:**

**1. Authority and responsibility**

1. Any suspected stroke patient with onset < 3 hours who arrives ED should be managed based on this protocol.
2. When a suspected stroke patient with onset < 3 hours is identified at the triage, the triage nursery should contact the ED physician at the critical care section immediately. The triage nursery should activate “Acute Stroke Protocol” by clicking a check box on the patient’s computerized order entry window and selecting the activation icon. The patient should be sent to the critical care section as soon as possible.
3. Any suspected stroke patient with onset < 3 hours should be evaluated by the critical care ED physician.
4. The “Acute Stroke Protocol” should be activated on any suspected stroke patient with onset < 3 hours, except those with confirmed hemorrhagic stroke or completely recovered transient ischemic attack.
5. The critical care nursery performs emergency blood test, including blood glucose measurement.
6. The critical care ED physician orders the head CT scan, and contacts the CT room with a specialized hotline (< 12 minutes).
7. The critical care ED physician consults the duty neurologist by telephone (< 15 minutes).
8. If the brain CT shows any hemorrhage, then consults the duty neurosurgeon.
9. If the brain CT shows no hemorrhage, then the neurologist should decide to perform thrombolysis or not.

**2. Standard operating procedures**

1. When a patients with acute neurological symptoms or consciousness change with onset < 3 hours arrives the ED, the triage nursery should contact the critical care ED physician immediately to evaluate whether this patient is a candidate for Acute Stroke Protocol. If the Emergency Medical System (EMS) uses a pre-hospital notification system to notify a suspected acute stroke patient with onset < 3 hours, then the patient should be sent directly into the critical care section instead of via triage.
2. If the patient is identified as a candidate, then the triage nursery could activate the “Acute Stroke Protocol” by clicking on the computerized triage interface; otherwise the ED physician could also activate the protocol via the diagnosis interface. The patient should be sent to the critical care section as soon as possible and be managed by the critical care ED physician.
3. The critical care ED physician evaluates the patient, orders the head CT scan and contacts the CT room by a specialized hotline (< 12 minutes).
4. The critical care nursery performs emergency blood test, including the OneTouch blood glucose measurement. The blood sample should be attached with a special mark showing “Acute Stroke Protocol”, and be sent to the laboratory room via the pneumatic tube system (< 17 minutes). The nursery should give the patient normal saline 500mL IV drip stat, and send the patient to the CT room.
5. The critical care nursery escort the patient to the CT room (< 15 minutes).
6. The critical care ED physician contacts the duty neurologist consultant by telephone (< 15 minutes).
7. The head CT scan is completed and uploaded toe the PACS system (< 25 minutes).
8. The duty neurologist arrives at bedside to assess the patient.
9. The laboratory department receives the blood sample with a special mark showing “Acute Stroke Protocol”, performs the blood test and uploads the data (< 60 minutes).
10. The duty neurologist confirms the patient as an acute ischemic stroke candidate for thrombolysis therapy. The ED physician and nursery should follow the duty neurologist’s instructions to transfer the patient to the stroke ICU for thrombolysis. If there is no bed available in the stroke ICU, the patient should be managed with thrombolysis at the critical care section.

**3. Quality control index:** The Stroke Center will document the following indexes and discuss the efficacy periodically.

1. The ED physician evaluates and completes the order of head CT scan and contact the CT room **within 12 minutes** (started from triage arrival time).
2. The ED physician consults the duty neurologist consultant **within 15 minutes** (started from triage arrival time).
3. The patient arrives the CT room **within 15 minutes** (started from triage arrival time).
4. The head CT scan is done **within 25 minutes** (started from triage arrival time, till the time the first scan is available on the PACS system).
5. The door-to-needle time **within 60 minutes** (started from triage arrival time, till the time first bolus injection of IV-tPA is given).

**Instruction and Informed Consent for Intravenous Thrombolysis Treatment**

This document is an introduction to the procedure you are about to receive. It will explain the process of the procedure, and its benefits, risks, and alternatives. Please read this document carefully. If you have any questions or concerns, please discuss them your physician. Our main goal is to work together to achieve an overall improvement in your health.

**Operation/Procedure:** Intravenous Thrombolytic Therapy Procedure.

**Benefits:** These are the possible benefits you might receive with this procedure. These benefits cannot be guaranteed by our Doctors. You will have to decide between the benefit/risk ratio of any medical procedure.

Your brain has experienced an ischemic stroke event, due to blood flow being restricted by a vessel blocked by a thrombus. There is a high probability of irreversible sequelae. If the thrombus can be rapidly resolved, there is a possibly of limiting the neurological damage. Intravenous tissue plasminogen activator (IV-tPA) can dissolve the thrombus clot efficiently and limit the damage of ischemic stroke. According to large-scale research performed by the NINDS, a group of patients receiving IV-tPA therapy had better outcomes than patients that did not receive therapy. A 3 month follow up of these patients showed that the patients that received the therapy improved chances of complete recovery and possibly less neurological damage. One large-scale European clinical study showed that compared with patients that did not receive thrombolytic therapy; patients that underwent the procedure had a better chance of improvement 3 months after the therapy.

**Risks:** No medical procedure is without risk. The following risks are well-studied in the clinical setting, but there is still the possibility of complications or risks that cannot be foreseen.

- 1. IV-tPA is currently the most beneficial therapy for an acute ischemic cerebral-vascular event, but the procedure is not without risk. In 100 patients that receive IV-TPA, 6 patients may be exposed to the risk of intracerebral hemorrhage. Hemorrhaging may worsen ischemic symptoms, and may progress to paralysis, loss of consciousness, and death. It may also cause bleeding elsewhere in the body.
  2. There is a 1% chance of an allergic reaction to tPA. Skin rash, bronchospasms, and/or vascular edema may occur.

**Alternatives:** These are the alternatives to this therapy. If you choose not to undergo this operation or procedure, please discuss the risks with your physician

IV-tPA therapy has been proven to be effective in large-scale clinical studies in the US and Europe. No other medication or treatment that has been proven to be as effective as IV-tPA. Your right to receive standardized medical care will not be compromised if you refuse to undergo this procedure. We will continue to administer standardized medical care of an acute ischemic cerebrovascular event.

Patients who are indicated for IV-tPA who do not receive the procedure may have a 30-50% worse outcome than patients who do receive IV-tPA.

All questions were answered and the patient/family/guardian consents to the procedure.

______________________________________M.D.

(Physician’s Signature)

DATE:___________________________________

Identification: I, __________________________________ (PATIENT), born on __________________ (DATE OF BIRTH), have suffered from Acute Ischemic Stroke, and am indicated for the Intravenous Thrombolytic Therapy. I have discussed with the physician and understood the benefits, risks, and alternatives of this procedure. I have read this form or have had it read to me, I understand this information and have no further questions.

I ☐ Agree for this procedure.

☐ Disagree

___________________________________________________________

(Patient’s/family’s/guardian’s signature *)

*(If patient’s signature cannot be obtained, indicate reason in comments section above)
