# Supplementary material for: Stroke Code Improves Intravenous Thrombolysis Administration in Acute Ischemic Stroke
Source: PLoS One. 2014 Aug 11;9(8):e104862. doi: 10.1371/journal.pone.0104862 (PMC4128738; doi:10.1371/journal.pone.0104862)
Supplement: Supporting Information S2 — Literature Review and Meta-analysis. (DOCX) [file pone.0104862.s004.docx]

**Supporting Information S2**

**Literature Review and Meta-analysis**

**Methods**

**The process of literature review**

Previous reports concerning hospitals implemented with programs to improve the performance of thrombolysis were reviewed. PubMed online search tools were applied to find related articles. Search entries were “door-to-needle time AND stroke”, or “door-to-needle time AND thrombolysis”, and were filtered by publication dates within 5 years (2009 – 2014) and written in English. Reports of thrombolysis performed for indications other than stroke were excluded. Additionally, the references of the selected articles were also checked to detect other eligible publications. Studies were selected only if they had a clear-cut comparison of the thrombolytic rate and/or functional outcome before and after any implemented strategy. In order to compare with our present study and to control factors such as learning effect, studies were excluded if their comparisons were based on the results from different strategies adopted during the same study period, not before and afterwards. Any study that duplicated another using the same study population was excluded from analysis. A meta-analysis was performed to determine the pooled odds ratio of increase of thrombolysis rate and good outcome after the implementation strategies.

**Results**

**Findings from previously reported studies and meta-analysis**

Using the search entries defined above, 95 studies were obtained from PubMed. Twelve studies were excluded because the indication of thrombolysis was not stroke and another one study was not using IV-tPA but mechanical thrombectomy. Another nine studies were excluded because they were reviews, expert opinions, or regional protocols. For the remaining 73 studies, 24 were hospital or registry reports regarding their thrombolysis performance without comparison, and were excluded from analysis. Another 29 studies were reporting comparison based on different strategies adopted during the same study period and were thus excluded. In the remaining 20 studies, 11 were not selected because they lacked of either thrombolysis rate or outcome data. The remaining 9 studies selected for the analysis were summarized in the Supplemental Table S2. Of these 9 studies, one was a citywide pre-hospital stroke program [15], another was a multi-hospital computerized program [19], and the remaining were all single hospital reports. All have documented thrombolysis rate (before and after the strategy implementation) and 6 studies have good functional outcome (mRS ≤2) data. A meta-analysis of these studies demonstrated a two-fold increase in chance of thrombolysis rate after the intervention (Figure 2). However, there was only a statistically nonsignificant trend toward good outcome (Figure 3).
